# The Apple Watch spO_2_ sensor and outliers in healthy users

**DOI:** 10.1038/s41746-023-00814-x

**Published:** 2023-04-08

**Authors:** Christina Schröder, Robert Förster, Daniel R. Zwahlen, Paul Windisch

**Affiliations:** Department of Radiation Oncology, Kantonsspital Winterthur, Winterthur, Switzerland

**Keywords:** Diagnostic markers, Biomarkers

## Abstract

It is unclear how frequently the Apple Watch produces spO_2_ measurements outside of the normal range in healthy individuals at rest. We conducted a head-to-head comparison in 38 healthy individuals between two watches and two medical-grade pulse oximeters. Fourteen percent of watch measurements yielded spO_2_ values below 95%, with no values below 92%. Results suggest that outliers measured by the watch should not be a cause for concern in otherwise healthy individuals.

Wearables are blurring the lines between lifestyle devices and medical products. Parameters that could only be measured using dedicated equipment in the past, are now available on many people’s wrist.

The electrocardiogram (ECG) function of the Apple Watch has been evaluated in large studies and received clearance by the United States Food and Drug Administration (FDA), while other features of the same device have not and are framed as wellness trackers^1^.

The spO_2_ measurement is one of these features, and previously published studies that compare spO_2_ measurements by the watch to those by a conventional, medical-grade pulse oximeter found no strong systematic bias between the two^2–6^.

This is somewhat surprising as the Apple Watch cannot use the common transmissive pulse oximetry, where light is passed through a thin part of the body, and instead has to rely on reflectance pulse oximetry, where light is passed into the wrist and only the reflected light is measured at the photodiode. This approach is considered more challenging as changes in spO_2_ tend to produce smaller changes in the signals, which also appear to be less stable^7^.

Since all of the previously mentioned studies investigated people with either cardiopulmonary conditions plus some healthy controls or healthy people in hypoxic conditions and only one of them shared patient-level data, the frequency and severity of outliers measured by the watch in people with actually healthy oxygen saturation in normoxic conditions remain difficult to assess.

This is an important metric since most people using an Apple Watch will be healthy and conspicuous values, outside of the normal range of 95–100% might cause distress to those who tend to worry a lot about their health.

The purpose of this study was, therefore, to assess the frequency of outliers in healthy participants as well as to share patient-level data that can be aggregated in future systematic reviews of the topic.

All the data collected in this study is available in supplementary table 1. The median age of participants was 32 years (range: 19–63 years). The most frequent Fitzpatrick skin type was 2 (55%), followed by 3 (21%), 4 (13%), 1 (8%), and 5 (3%). The median arm circumference at the measurement site was 16 cm (range: 14–20.5 cm). Two participants reported a history of pneumonia and another one mentioned having asthma, while the remaining 35 did not report any history of lung disease. No skin irregularities on the wrist were noted.

The mean difference between the conventional pulse oximeter measurements in the same participant was 1.39% spO_2_ (range: 0–4% spO_2_). The mean difference between the two Apple Watch measurements in the same patient was 2.35% spO_2_ (range: 0–6% spO_2_). The mean heart rate was 69 bpm (range: 41–101 bpm).

Of the 76 Apple Watch measurements, 28 (36%) were preceded by at least one failed measurement. In one participant, one of the watches could not obtain a measurement after using a maximum of three attempts. While only one measurement using a conventional pulse oximeter resulted in a value below 95%, there were eleven (14%) Apple Watch measurements below 95%, including two that measured a spO_2_ of 92% (Fig. 1).

**Fig. 1:**
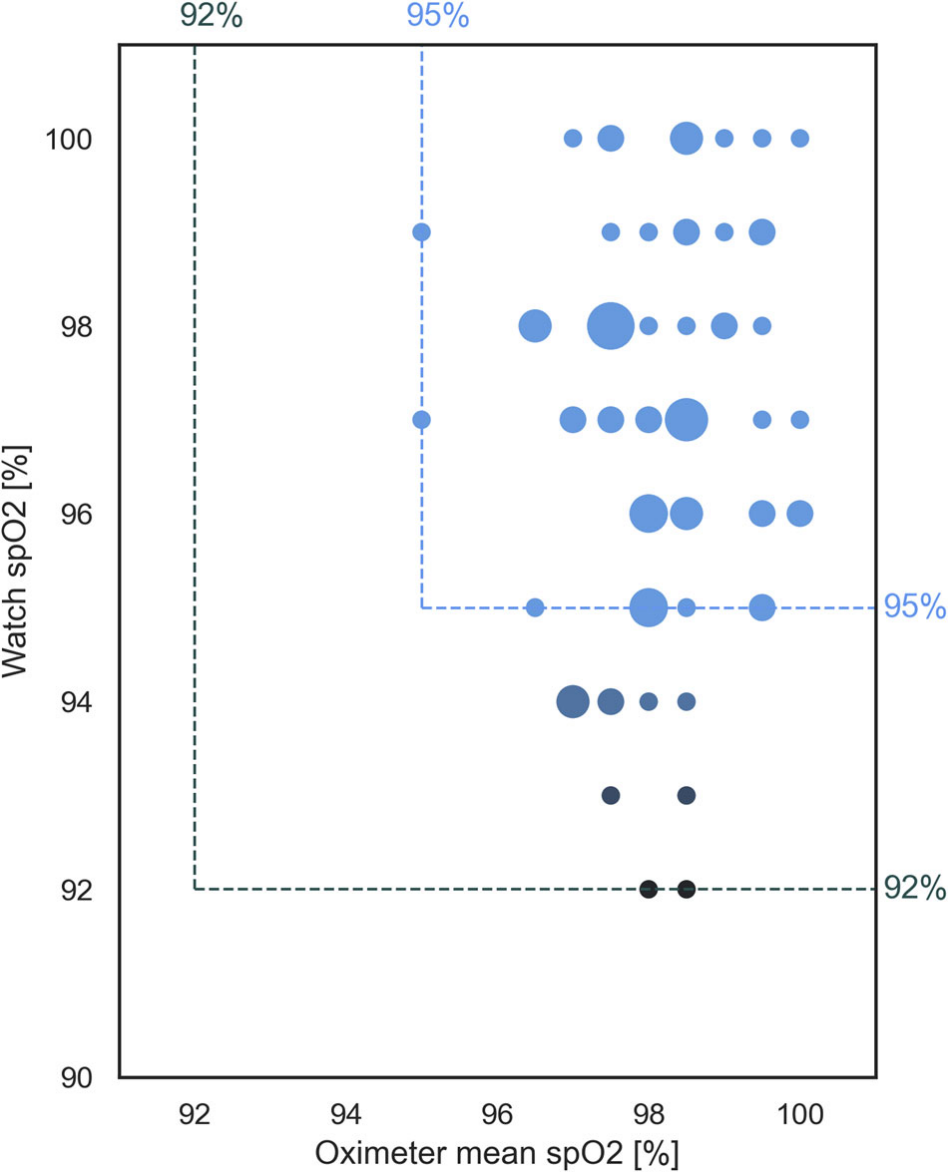
Scatter plot of spO_2_ measurements. Each dot represents a measurement with an Apple Watch. The y-coordinate is the result of that measurement, and the x-coordinate is the mean of the conventional pulse oximeter measurements of the same participant. If several measurements share the same location, this is indicated by the size of the dot, with the biggest dots representing six measurements.

Our study demonstrates that spO_2_ measurements by the Apple Watch that fall outside of the 95–100% range are a fairly common occurrence in healthy participants at rest. Therefore, isolated conspicuous measurements in otherwise healthy individuals should not be a cause for concern, while a chronic reduction in the average spO_2_ measured by the watch might be a more relevant indication of an underlying issue.

While we did not see outliers below 92%, it is unclear if these can also happen, although rarely, or if values below 92% are indicative of true hypoxemia.

As most people wearing an Apple Watch are regular “users” and not “patients”, a value outside of what the results of an internet search refer to as normal, might cause distress and unnecessary visits to their healthcare providers. Therefore, even if isolated values between 92 and 95% spO_2_ at rest would probably not be considered clinically relevant by most physicians, they can become relevant to someone’s wellbeing and the healthcare system due to people not having the same knowledge.

A previous publication by Paetz and colleagues demonstrated that the way the watch was worn greatly influenced the number of successful measurements, which poses the question if the number of outliers could potentially be higher when the watch doesn’t measure under study conditions but when the person wearing it is just going about their day.

Even though the watch occasionally produces outliers, there might still be value to using it in remote monitoring settings in the future as long as the focus of these settings is not on robustly identifying sudden drops in spO_2_, but on following changes of the average spO_2_ over longer periods of time.

Another question that warrants further research is the frequency of outliers in people with varying skin colors. Studies with conventional pulse oximeters have already demonstrated different risks of occult hypoxemia for different skin colors^8,9^. Our study was not powered to predict the frequency of outliers by skin color; however, the Fitzpatrick skin type was collected and is provided in Supplementary Table 1.

This study is limited by its sample size as well as the fact that the Apple Watches were both from series 6 and that the performance of the most recent model might differ. In addition, we used conventional, medical-grade pulse oximeters as the ground truth, which may also show some inaccuracies compared to arterial blood samples.

In conclusion, the Apple Watch frequently measures spO_2_ values outside of the 95–100% range despite the fact that the watch is worn by healthy participants at rest. Outliers measured by the watch should not be a cause for concern in otherwise healthy individuals as long as the average spO_2_ remains stable. Questions regarding the impact of other parameters, such as wrist circumference, skin color, etc., on the frequency and severity of outliers, remain to be answered.

## Methods

Thirty-eight healthy volunteers working in our department were included. Participants were asked ahead of the study to participate via email and, after obtaining written informed consent, were instructed to sit down in a comfortable position while two authors (C.S. and P.W.) collected the participant characteristics, including age, Fitzpatrick skin type, and self-reported history of lung disease. In addition, the circumference and any skin abnormalities of the left wrist were noted.

After at least 1 min had passed, the participants were measured at 440 m above mean sea level using two Apple Watches (Apple Watch Series 6, Apple Inc., Cupertino, CA) updated to the latest available software version and two conventional pulse oximeters (GE Healthcare V100, GE Healthcare, Chicago, IL) respectively. All measurements were conducted in immediate succession on the left ring finger and the left wrist of the respective participants, while the order of measurements was randomly decided for each participant using an online tool^10^. Two official wristbands were used and switched between the watches after a batch of ten participants had been measured.

Participants were instructed to place the watch on their left wrist according to the official documentation: “*The band should be snug but comfortable, and the back of your Apple Watch needs to be touching your wrist*”^11^.

The measurements with conventional pulse oximeters were conducted using the ring finger on the same hand. During all measurements, participants were instructed to breathe normally while not talking and keeping their left arm relaxed on a table in front of them with the watch display facing up. If a measurement with an Apple Watch was unsuccessful, patients were instructed to readjust the watch. A maximum of three measurements per watch was attempted. The heart rate was noted during the first measurement with a conventional pulse oximeter.

The study design was evaluated by an ethics commission that decided that the study did not require authorization as long as it served only as an orientation without attempting to make definitive statements on the accuracy of the Apple Watch for which it is not powered (Kantonale Ethikkommission, BASEC-Nr. Req-2021-01068).

### Reporting summary

Further information on research design is available in the Nature Research Reporting Summary linked to this article.

## Supplementary information

Supplementary Information

REPORTING SUMMARY

Supp Data

## Data Availability

The full dataset that was created and used as part of this study is provided in Supplementary Table 1.
