# Supplementary material for: The Apple Watch spO2 sensor and outliers in healthy users
Source: NPJ Digit Med. 2023 Apr 8;6:63. doi: 10.1038/s41746-023-00814-x (PMC10082785; doi:10.1038/s41746-023-00814-x)
Supplement: Supplementary file 1 — Supplementary Information [file 41746_2023_814_MOESM1_ESM.pdf]

Supplementary Table 1. The full participant-level dataset obtained as part of the study.
